# Supplementary material for: Differential personality change earlier and later in the coronavirus pandemic in a longitudinal sample of adults in the United States
Source: PLoS One. 2022 Sep 28;17(9):e0274542. doi: 10.1371/journal.pone.0274542 (PMC9518853; doi:10.1371/journal.pone.0274542)
Supplement: S1 Table — (DOCX) [file pone.0274542.s001.docx]

Supplemental Table S1

*Interaction terms between sociodemographic factors and the pandemic on personality change*

| Model estimates |  | Neuroticism | Extraversion | Openness | Agreeableness | Conscientiousness |
| --- | --- | --- | --- | --- | --- | --- |
| Gender |  |  |  |  |  |  |
| 2020 |  | -.28 (.12) | .26 (.10)** | -.04 (.11) | .13 (.11) | .16 (.11) |
| 2021 |  | -.27 (.20) | .27 (.18) | .06 (.20) | .39 (.19) | .24 (.19) |
| Age |  |  |  |  |  |  |
| 2020 |  | -.02 (.004)** | .01 (.00) | .00 (.00) | .01 (.003)** | .01 (.003) |
| 2021 |  | -.02 (.01)** | .01 (.005) | .01 (.006) | .02 (.005)** | .02 (.005)** |
| Race (Black) |  |  |  |  |  |  |
| 2020 |  | .24 (.21) | -.12 (.18) | -.11 (.20) | .12 (.20) | -.23 (.19) |
| 2021 |  | .46 (.36) | -.28 (.31) | -.06 (.35) | -.33 (.34) | -.44 (.33) |
| Race (Asian) |  |  |  |  |  |  |
| 2020 |  | .30 (.31) | -.04 (.27) | -.03 (.30) | .04 (.30) | .26 (.29) |
| 2021 |  | .46 (.36) | -.16 (.31) | .34 (.35) | -.07 (.34) | .71 (.33) |
| Race (Otherwise identified) |  |  |  |  |  |  |
| 2020 |  | .11 (.21) | -.08 (.18) | -.15 (.20) | -.24 (.20) | -.47 (.19) |
| 2021 |  | .34 (.32) | -.50 (.28) | -.54 (.31) | -.86 (.30)** | -.61 (.29) |
| Hispanic/Latino ethnicity |  |  |  |  |  |  |
| 2020 |  | .73 (.17)** | -.35 (.15) | -.30 (.17) | -.44 (.16)** | -.26 (.16) |
| 2021 |  | .46 (.22) | -.73 (.19)** | -.62 (.21)** | -.33 (.21) | -.59 (.20)** |
| Education |  |  |  |  |  |  |
| 2020 |  | -.02 (.02) | .00 (.02) | -.02 (.02) | .02 (.02) | .04 (.02) |
| 2021 |  | .02 (.04) | .10 (.04) | .06 (.04) | .10 (.04) | .07 (.04) |

*Note*. The one significant interaction for gender indicated a decline in extraversion in 2020 compared to pre-pandemic levels for women but not men. The one significant interaction for race indicated that Otherwise identified participants had a greater decline in Agreeableness in 2021-2022 compared to white participants. Note that this interaction is difficult to interpret because of the diversity of this group. The interactions with age and Hispanic/Latino ethnicity are described in the Results.

***p*<.01.
