# Supplementary material for: Differential personality change earlier and later in the coronavirus pandemic in a longitudinal sample of adults in the United States
Source: PLoS One. 2022 Sep 28;17(9):e0274542. doi: 10.1371/journal.pone.0274542 (PMC9518853; doi:10.1371/journal.pone.0274542)
Supplement: S2 Table — (DOCX) [file pone.0274542.s002.docx]

Supplemental Material: Syntax

**Note: copy and paste into SPSS syntax to run with the datafile**

**Compute time**

IF (Index1 = 1 & not missing(DATE)) time=0.

IF (Index1 = 2 & not missing(DATE)) time=0.

IF (Index1 = 3 & not missing(DATE)) time=0.

EXECUTE.

IF (uasid = LAG(uasid) & Index1 = 2 & not missing(LAG(DATE))) time=DATEDIFF(date,LAG(date),"days")/365.25.

IF (uasid = LAG(uasid,2) & Index1 = 3) time=DATEDIFF(date,LAG(date,2),"days")/365.25.

EXECUTE.

IF (uasid = LAG(uasid,2) & Index1 = 3 & missing(LAG(DATE,2))) & not missing(LAG(DATE)) time=DATEDIFF(date,LAG(date),"days")/365.25.

EXECUTE.

IF (Index1 = 3 & DATE > 0 & missing(LAG(DATE,2)) & missing(LAG(DATE))) time=0.

EXECUTE.

*COVID codes assessment during COVID, which in this sample was before the date on March 1st.

COMPUTE COVID.date=DATE.MDY(03,01,2020).

EXECUTE.

IF (DATE >= COVID.date) covid=1.

IF (DATE < COVID.date) covid=0.

EXECUTE.

**calculate by period of the pandemic**

COMPUTE COVID.date2020march=DATE.MDY(03,01,2020).

COMPUTE COVID.date2020dic=DATE.MDY(12,31,2020).

EXECUTE.

IF (DATE >= COVID.date2020march & DATE <= COVID.date2020dic) covid2020=1.

IF (DATE > COVID.date2020dic) covid2020=0.

IF (DATE < COVID.date2020march) covid2020=0.

IF (DATE > COVID.date2020dic) covid2021=1.

IF (DATE <= COVID.date2020dic) covid2021=0.

EXECUTE.

**Calculate the longitudinal sample**

SORT CASES BY uasid(A) Index1(A).

IF (uasid = LAG(uasid) & covid = 1 & LAG(covid) = 1) longitudinal=2.

IF (uasid = LAG(uasid,2) & covid = 1 & LAG(covid,2) = 0) longitudinal=1.

IF (uasid = LAG(uasid) & covid = 1 & LAG(covid) = 0) longitudinal=1.

EXECUTE.

SORT CASES BY uasid(A) Index1(D).

IF (uasid = LAG(uasid) AND not missing(covid)) longitudinal=LAG(longitudinal).

IF (uasid = LAG(uasid,2) AND not missing(covid)) longitudinal=LAG(longitudinal,2).

EXECUTE.

SORT CASES BY uasid(A) Index1(A).

**Select longitudinal cases**

USE ALL.

COMPUTE filter_$=(longitudinal = 1).

VARIABLE LABELS filter_$ 'longitudinal = 1 (FILTER)'.

VALUE LABELS filter_$ 0 'Not Selected' 1 'Selected'.

FORMATS filter_$ (f1.0).

FILTER BY filter_$.

EXECUTE.

MIXED N WITH age gender education race_d1 race_d2 race_d3 Hisp time covid2020 covid2021

/CRITERIA=DFMETHOD(SATTERTHWAITE) CIN(95) MXITER(100) MXSTEP(10) SCORING(1)

SINGULAR(0.000000000001) HCONVERGE(0, ABSOLUTE) LCONVERGE(0, ABSOLUTE) PCONVERGE(0.000001, ABSOLUTE)

/FIXED=age gender education race_d1 race_d2 race_d3 Hisp time covid2020 covid2021 | SSTYPE(3)

/METHOD=ML

/PRINT=DESCRIPTIVES SOLUTION TESTCOV

/RANDOM=INTERCEPT time | SUBJECT(uasid) COVTYPE(VC).

MIXED E WITH age gender education race_d1 race_d2 race_d3 Hisp time covid2020 covid2021

/CRITERIA=DFMETHOD(SATTERTHWAITE) CIN(95) MXITER(100) MXSTEP(10) SCORING(1)

SINGULAR(0.000000000001) HCONVERGE(0, ABSOLUTE) LCONVERGE(0, ABSOLUTE) PCONVERGE(0.000001, ABSOLUTE)

/FIXED=age gender education race_d1 race_d2 race_d3 Hisp time covid2020 covid2021 | SSTYPE(3)

/METHOD=ML

/PRINT=DESCRIPTIVES SOLUTION TESTCOV

/RANDOM=INTERCEPT time | SUBJECT(uasid) COVTYPE(VC).

MIXED O WITH age gender education race_d1 race_d2 race_d3 Hisp time covid2020 covid2021

/CRITERIA=DFMETHOD(SATTERTHWAITE) CIN(95) MXITER(100) MXSTEP(10) SCORING(1)

SINGULAR(0.000000000001) HCONVERGE(0, ABSOLUTE) LCONVERGE(0, ABSOLUTE) PCONVERGE(0.000001, ABSOLUTE)

/FIXED=age gender education race_d1 race_d2 race_d3 Hisp time covid2020 covid2021 | SSTYPE(3)

/METHOD=ML

/PRINT=DESCRIPTIVES SOLUTION TESTCOV

/RANDOM=INTERCEPT time | SUBJECT(uasid) COVTYPE(VC).

MIXED A WITH age gender education race_d1 race_d2 race_d3 Hisp time covid2020 covid2021

/CRITERIA=DFMETHOD(SATTERTHWAITE) CIN(95) MXITER(100) MXSTEP(10) SCORING(1)

SINGULAR(0.000000000001) HCONVERGE(0, ABSOLUTE) LCONVERGE(0, ABSOLUTE) PCONVERGE(0.000001, ABSOLUTE)

/FIXED=age gender education race_d1 race_d2 race_d3 Hisp time covid2020 covid2021 | SSTYPE(3)

/METHOD=ML

/PRINT=DESCRIPTIVES SOLUTION TESTCOV

/RANDOM=INTERCEPT time | SUBJECT(uasid) COVTYPE(VC).

MIXED C WITH age gender education race_d1 race_d2 race_d3 Hisp time covid2020 covid2021

/CRITERIA=DFMETHOD(SATTERTHWAITE) CIN(95) MXITER(100) MXSTEP(10) SCORING(1)

SINGULAR(0.000000000001) HCONVERGE(0, ABSOLUTE) LCONVERGE(0, ABSOLUTE) PCONVERGE(0.000001, ABSOLUTE)

/FIXED=age gender education race_d1 race_d2 race_d3 Hisp time covid2020 covid2021 | SSTYPE(3)

/METHOD=ML

/PRINT=DESCRIPTIVES SOLUTION TESTCOV

/RANDOM=INTERCEPT time | SUBJECT(uasid) COVTYPE(VC).

**Analysis by age group**

SORT CASES BY age@.

SPLIT FILE LAYERED BY age@.

MIXED N WITH age gender education race_d1 race_d2 race_d3 Hisp time covid2020 covid2021

/CRITERIA=DFMETHOD(SATTERTHWAITE) CIN(95) MXITER(100) MXSTEP(10) SCORING(1)

SINGULAR(0.000000000001) HCONVERGE(0, ABSOLUTE) LCONVERGE(0, ABSOLUTE) PCONVERGE(0.000001, ABSOLUTE)

/FIXED=age gender education race_d1 race_d2 race_d3 Hisp time covid2020 covid2021 | SSTYPE(3)

/METHOD=ML

/PRINT=DESCRIPTIVES SOLUTION TESTCOV

/RANDOM=INTERCEPT time | SUBJECT(uasid) COVTYPE(VC).

MIXED E WITH age gender education race_d1 race_d2 race_d3 Hisp time covid2020 covid2021

/CRITERIA=DFMETHOD(SATTERTHWAITE) CIN(95) MXITER(100) MXSTEP(10) SCORING(1)

SINGULAR(0.000000000001) HCONVERGE(0, ABSOLUTE) LCONVERGE(0, ABSOLUTE) PCONVERGE(0.000001, ABSOLUTE)

/FIXED=age gender education race_d1 race_d2 race_d3 Hisp time covid2020 covid2021 | SSTYPE(3)

/METHOD=ML

/PRINT=DESCRIPTIVES SOLUTION TESTCOV

/RANDOM=INTERCEPT time | SUBJECT(uasid) COVTYPE(VC).

MIXED O WITH age gender education race_d1 race_d2 race_d3 Hisp time covid2020 covid2021

/CRITERIA=DFMETHOD(SATTERTHWAITE) CIN(95) MXITER(100) MXSTEP(10) SCORING(1)

SINGULAR(0.000000000001) HCONVERGE(0, ABSOLUTE) LCONVERGE(0, ABSOLUTE) PCONVERGE(0.000001, ABSOLUTE)

/FIXED=age gender education race_d1 race_d2 race_d3 Hisp time covid2020 covid2021 | SSTYPE(3)

/METHOD=ML

/PRINT=DESCRIPTIVES SOLUTION TESTCOV

/RANDOM=INTERCEPT time | SUBJECT(uasid) COVTYPE(VC).

MIXED A WITH age gender education race_d1 race_d2 race_d3 Hisp time covid2020 covid2021

/CRITERIA=DFMETHOD(SATTERTHWAITE) CIN(95) MXITER(100) MXSTEP(10) SCORING(1)

SINGULAR(0.000000000001) HCONVERGE(0, ABSOLUTE) LCONVERGE(0, ABSOLUTE) PCONVERGE(0.000001, ABSOLUTE)

/FIXED=age gender education race_d1 race_d2 race_d3 Hisp time covid2020 covid2021 | SSTYPE(3)

/METHOD=ML

/PRINT=DESCRIPTIVES SOLUTION TESTCOV

/RANDOM=INTERCEPT time | SUBJECT(uasid) COVTYPE(VC).

MIXED C WITH age gender education race_d1 race_d2 race_d3 Hisp time covid2020 covid2021

/CRITERIA=DFMETHOD(SATTERTHWAITE) CIN(95) MXITER(100) MXSTEP(10) SCORING(1)

SINGULAR(0.000000000001) HCONVERGE(0, ABSOLUTE) LCONVERGE(0, ABSOLUTE) PCONVERGE(0.000001, ABSOLUTE)

/FIXED=age gender education race_d1 race_d2 race_d3 Hisp time covid2020 covid2021 | SSTYPE(3)

/METHOD=ML

/PRINT=DESCRIPTIVES SOLUTION TESTCOV

/RANDOM=INTERCEPT time | SUBJECT(uasid) COVTYPE(VC).

**Interactions with age**

MIXED N WITH age_cent gender race_d1 race_d2 race_d3 Hisp education time covid2020 covid2021 covid20_age covid21_age

/CRITERIA=DFMETHOD(SATTERTHWAITE) CIN(95) MXITER(100) MXSTEP(10) SCORING(1)

SINGULAR(0.000000000001) HCONVERGE(0, ABSOLUTE) LCONVERGE(0, ABSOLUTE) PCONVERGE(0.000001, ABSOLUTE)

/FIXED=age_cent gender race_d1 race_d2 race_d3 Hisp education time covid2020 covid2021 covid20_age covid21_age | SSTYPE(3)

/METHOD=ML

/PRINT=DESCRIPTIVES SOLUTION TESTCOV

/RANDOM=INTERCEPT time | SUBJECT(uasid) COVTYPE(VC).

MIXED E WITH age_cent gender race_d1 race_d2 race_d3 Hisp education time covid2020 covid2021 covid20_age covid21_age

/CRITERIA=DFMETHOD(SATTERTHWAITE) CIN(95) MXITER(100) MXSTEP(10) SCORING(1)

SINGULAR(0.000000000001) HCONVERGE(0, ABSOLUTE) LCONVERGE(0, ABSOLUTE) PCONVERGE(0.000001, ABSOLUTE)

/FIXED=age_cent gender race_d1 race_d2 race_d3 Hisp education time covid2020 covid2021 covid20_age covid21_age | SSTYPE(3)

/METHOD=ML

/PRINT=DESCRIPTIVES SOLUTION TESTCOV

/RANDOM=INTERCEPT time | SUBJECT(uasid) COVTYPE(VC).

MIXED O WITH age_cent gender race_d1 race_d2 race_d3 Hisp education time covid2020 covid2021 covid20_age covid21_age

/CRITERIA=DFMETHOD(SATTERTHWAITE) CIN(95) MXITER(100) MXSTEP(10) SCORING(1)

SINGULAR(0.000000000001) HCONVERGE(0, ABSOLUTE) LCONVERGE(0, ABSOLUTE) PCONVERGE(0.000001, ABSOLUTE)

/FIXED=age_cent gender race_d1 race_d2 race_d3 Hisp education time covid2020 covid2021 covid20_age covid21_age | SSTYPE(3)

/METHOD=ML

/PRINT=DESCRIPTIVES SOLUTION TESTCOV

/RANDOM=INTERCEPT time | SUBJECT(uasid) COVTYPE(VC).

MIXED A WITH age_cent gender race_d1 race_d2 race_d3 Hisp education time covid2020 covid2021 covid20_age covid21_age

/CRITERIA=DFMETHOD(SATTERTHWAITE) CIN(95) MXITER(100) MXSTEP(10) SCORING(1)

SINGULAR(0.000000000001) HCONVERGE(0, ABSOLUTE) LCONVERGE(0, ABSOLUTE) PCONVERGE(0.000001, ABSOLUTE)

/FIXED=age_cent gender race_d1 race_d2 race_d3 Hisp education time covid2020 covid2021 covid20_age covid21_age | SSTYPE(3)

/METHOD=ML

/PRINT=DESCRIPTIVES SOLUTION TESTCOV

/RANDOM=INTERCEPT time | SUBJECT(uasid) COVTYPE(VC).

MIXED C WITH age_cent gender race_d1 race_d2 race_d3 Hisp education time covid2020 covid2021 covid20_age covid21_age

/CRITERIA=DFMETHOD(SATTERTHWAITE) CIN(95) MXITER(100) MXSTEP(10) SCORING(1)

SINGULAR(0.000000000001) HCONVERGE(0, ABSOLUTE) LCONVERGE(0, ABSOLUTE) PCONVERGE(0.000001, ABSOLUTE)

/FIXED=age_cent gender race_d1 race_d2 race_d3 Hisp education time covid2020 covid2021 covid20_age covid21_age | SSTYPE(3)

/METHOD=ML

/PRINT=DESCRIPTIVES SOLUTION TESTCOV

/RANDOM=INTERCEPT time | SUBJECT(uasid) COVTYPE(VC).

**Interactions with gender**

MIXED N WITH age gender education race_d1 race_d2 race_d3 Hisp time covid2020 covid2021 covid20_gender covid21_gender

/CRITERIA=DFMETHOD(SATTERTHWAITE) CIN(95) MXITER(100) MXSTEP(10) SCORING(1)

SINGULAR(0.000000000001) HCONVERGE(0, ABSOLUTE) LCONVERGE(0, ABSOLUTE) PCONVERGE(0.000001, ABSOLUTE)

/FIXED=age gender education race_d1 race_d2 race_d3 Hisp time covid2020 covid2021 covid20_gender covid21_gender| SSTYPE(3)

/METHOD=ML

/PRINT=DESCRIPTIVES SOLUTION TESTCOV

/RANDOM=INTERCEPT time | SUBJECT(uasid) COVTYPE(VC).

MIXED E WITH age gender education race_d1 race_d2 race_d3 Hisp time covid2020 covid2021 covid20_gender covid21_gender

/CRITERIA=DFMETHOD(SATTERTHWAITE) CIN(95) MXITER(100) MXSTEP(10) SCORING(1)

SINGULAR(0.000000000001) HCONVERGE(0, ABSOLUTE) LCONVERGE(0, ABSOLUTE) PCONVERGE(0.000001, ABSOLUTE)

/FIXED=age gender education race_d1 race_d2 race_d3 Hisp time covid2020 covid2021 covid20_gender covid21_gender| SSTYPE(3)

/METHOD=ML

/PRINT=DESCRIPTIVES SOLUTION TESTCOV

/RANDOM=INTERCEPT time | SUBJECT(uasid) COVTYPE(VC).

MIXED O WITH age gender education race_d1 race_d2 race_d3 Hisp time covid2020 covid2021 covid20_gender covid21_gender

/CRITERIA=DFMETHOD(SATTERTHWAITE) CIN(95) MXITER(100) MXSTEP(10) SCORING(1)

SINGULAR(0.000000000001) HCONVERGE(0, ABSOLUTE) LCONVERGE(0, ABSOLUTE) PCONVERGE(0.000001, ABSOLUTE)

/FIXED=age gender education race_d1 race_d2 race_d3 Hisp time covid2020 covid2021 covid20_gender covid21_gender| SSTYPE(3)

/METHOD=ML

/PRINT=DESCRIPTIVES SOLUTION TESTCOV

/RANDOM=INTERCEPT time | SUBJECT(uasid) COVTYPE(VC).

MIXED A WITH age gender education race_d1 race_d2 race_d3 Hisp time covid2020 covid2021 covid20_gender covid21_gender

/CRITERIA=DFMETHOD(SATTERTHWAITE) CIN(95) MXITER(100) MXSTEP(10) SCORING(1)

SINGULAR(0.000000000001) HCONVERGE(0, ABSOLUTE) LCONVERGE(0, ABSOLUTE) PCONVERGE(0.000001, ABSOLUTE)

/FIXED=age gender education race_d1 race_d2 race_d3 Hisp time covid2020 covid2021 covid20_gender covid21_gender| SSTYPE(3)

/METHOD=ML

/PRINT=DESCRIPTIVES SOLUTION TESTCOV

/RANDOM=INTERCEPT time | SUBJECT(uasid) COVTYPE(VC).

MIXED C WITH age gender education race_d1 race_d2 race_d3 Hisp time covid2020 covid2021 covid20_gender covid21_gender

/CRITERIA=DFMETHOD(SATTERTHWAITE) CIN(95) MXITER(100) MXSTEP(10) SCORING(1)

SINGULAR(0.000000000001) HCONVERGE(0, ABSOLUTE) LCONVERGE(0, ABSOLUTE) PCONVERGE(0.000001, ABSOLUTE)

/FIXED=age gender education race_d1 race_d2 race_d3 Hisp time covid2020 covid2021 covid20_gender covid21_gender| SSTYPE(3)

/METHOD=ML

/PRINT=DESCRIPTIVES SOLUTION TESTCOV

/RANDOM=INTERCEPT time | SUBJECT(uasid) COVTYPE(VC).

**Interaction with race**

MIXED N WITH age gender race_d1 race_d2 race_d3 Hisp education time covid2020 covid2021 covid20_d1 covid20_d2 covid20_d3 covid21_d1 covid21_d2 covid21_d3

/CRITERIA=DFMETHOD(SATTERTHWAITE) CIN(95) MXITER(100) MXSTEP(10) SCORING(1)

SINGULAR(0.000000000001) HCONVERGE(0, ABSOLUTE) LCONVERGE(0, ABSOLUTE) PCONVERGE(0.000001, ABSOLUTE)

/FIXED=age gender race_d1 race_d2 race_d3 Hisp education time covid2020 covid2021 covid20_d1 covid20_d2 covid20_d3 covid21_d1 covid21_d2 covid21_d3 | SSTYPE(3)

/METHOD=ML

/PRINT=DESCRIPTIVES SOLUTION TESTCOV

/RANDOM=INTERCEPT time | SUBJECT(uasid) COVTYPE(VC).

MIXED E WITH age gender race_d1 race_d2 race_d3 Hisp education time covid2020 covid2021 covid20_d1 covid20_d2 covid20_d3 covid21_d1 covid21_d2 covid21_d3

/CRITERIA=DFMETHOD(SATTERTHWAITE) CIN(95) MXITER(100) MXSTEP(10) SCORING(1)

SINGULAR(0.000000000001) HCONVERGE(0, ABSOLUTE) LCONVERGE(0, ABSOLUTE) PCONVERGE(0.000001, ABSOLUTE)

/FIXED=age gender race_d1 race_d2 race_d3 Hisp education time covid2020 covid2021 covid20_d1 covid20_d2 covid20_d3 covid21_d1 covid21_d2 covid21_d3 | SSTYPE(3)

/METHOD=ML

/PRINT=DESCRIPTIVES SOLUTION TESTCOV

/RANDOM=INTERCEPT time | SUBJECT(uasid) COVTYPE(VC).

MIXED O WITH age gender race_d1 race_d2 race_d3 Hisp education time covid2020 covid2021 covid20_d1 covid20_d2 covid20_d3 covid21_d1 covid21_d2 covid21_d3

/CRITERIA=DFMETHOD(SATTERTHWAITE) CIN(95) MXITER(100) MXSTEP(10) SCORING(1)

SINGULAR(0.000000000001) HCONVERGE(0, ABSOLUTE) LCONVERGE(0, ABSOLUTE) PCONVERGE(0.000001, ABSOLUTE)

/FIXED=age gender race_d1 race_d2 race_d3 Hisp education time covid2020 covid2021 covid20_d1 covid20_d2 covid20_d3 covid21_d1 covid21_d2 covid21_d3 | SSTYPE(3)

/METHOD=ML

/PRINT=DESCRIPTIVES SOLUTION TESTCOV

/RANDOM=INTERCEPT time | SUBJECT(uasid) COVTYPE(VC).

MIXED A WITH age gender race_d1 race_d2 race_d3 Hisp education time covid2020 covid2021 covid20_d1 covid20_d2 covid20_d3 covid21_d1 covid21_d2 covid21_d3

/CRITERIA=DFMETHOD(SATTERTHWAITE) CIN(95) MXITER(100) MXSTEP(10) SCORING(1)

SINGULAR(0.000000000001) HCONVERGE(0, ABSOLUTE) LCONVERGE(0, ABSOLUTE) PCONVERGE(0.000001, ABSOLUTE)

/FIXED=age gender race_d1 race_d2 race_d3 Hisp education time covid2020 covid2021 covid20_d1 covid20_d2 covid20_d3 covid21_d1 covid21_d2 covid21_d3 | SSTYPE(3)

/METHOD=ML

/PRINT=DESCRIPTIVES SOLUTION TESTCOV

/RANDOM=INTERCEPT time | SUBJECT(uasid) COVTYPE(VC).

MIXED C WITH age gender race_d1 race_d2 race_d3 Hisp education time covid2020 covid2021 covid20_d1 covid20_d2 covid20_d3 covid21_d1 covid21_d2 covid21_d3

/CRITERIA=DFMETHOD(SATTERTHWAITE) CIN(95) MXITER(100) MXSTEP(10) SCORING(1)

SINGULAR(0.000000000001) HCONVERGE(0, ABSOLUTE) LCONVERGE(0, ABSOLUTE) PCONVERGE(0.000001, ABSOLUTE)

/FIXED=age gender race_d1 race_d2 race_d3 Hisp education time covid2020 covid2021 covid20_d1 covid20_d2 covid20_d3 covid21_d1 covid21_d2 covid21_d3 | SSTYPE(3)

/METHOD=ML

/PRINT=DESCRIPTIVES SOLUTION TESTCOV

/RANDOM=INTERCEPT time | SUBJECT(uasid) COVTYPE(VC).

**Interactions with ethnicity**

MIXED N WITH age gender education race_d1 race_d2 race_d3 hisp time covid2020 covid2021 covid20_hisp covid21_hisp

/CRITERIA=DFMETHOD(SATTERTHWAITE) CIN(95) MXITER(100) MXSTEP(10) SCORING(1)

SINGULAR(0.000000000001) HCONVERGE(0, ABSOLUTE) LCONVERGE(0, ABSOLUTE) PCONVERGE(0.000001, ABSOLUTE)

/FIXED=age gender education race_d1 race_d2 race_d3 hisp time covid2020 covid2021 covid20_hisp covid21_hisp | SSTYPE(3)

/METHOD=ML

/PRINT=DESCRIPTIVES SOLUTION TESTCOV

/RANDOM=INTERCEPT time | SUBJECT(uasid) COVTYPE(VC).

MIXED E WITH age gender education race_d1 race_d2 race_d3 hisp time covid2020 covid2021 covid20_hisp covid21_hisp

/CRITERIA=DFMETHOD(SATTERTHWAITE) CIN(95) MXITER(100) MXSTEP(10) SCORING(1)

SINGULAR(0.000000000001) HCONVERGE(0, ABSOLUTE) LCONVERGE(0, ABSOLUTE) PCONVERGE(0.000001, ABSOLUTE)

/FIXED=age gender education race_d1 race_d2 race_d3 hisp time covid2020 covid2021 covid20_hisp covid21_hisp | SSTYPE(3)

/METHOD=ML

/PRINT=DESCRIPTIVES SOLUTION TESTCOV

/RANDOM=INTERCEPT time | SUBJECT(uasid) COVTYPE(VC).

MIXED O WITH age gender education race_d1 race_d2 race_d3 hisp time covid2020 covid2021 covid20_hisp covid21_hisp

/CRITERIA=DFMETHOD(SATTERTHWAITE) CIN(95) MXITER(100) MXSTEP(10) SCORING(1)

SINGULAR(0.000000000001) HCONVERGE(0, ABSOLUTE) LCONVERGE(0, ABSOLUTE) PCONVERGE(0.000001, ABSOLUTE)

/FIXED=age gender education race_d1 race_d2 race_d3 hisp time covid2020 covid2021 covid20_hisp covid21_hisp | SSTYPE(3)

/METHOD=ML

/PRINT=DESCRIPTIVES SOLUTION TESTCOV

/RANDOM=INTERCEPT time | SUBJECT(uasid) COVTYPE(VC).

MIXED A WITH age gender education race_d1 race_d2 race_d3 hisp time covid2020 covid2021 covid20_hisp covid21_hisp

/CRITERIA=DFMETHOD(SATTERTHWAITE) CIN(95) MXITER(100) MXSTEP(10) SCORING(1)

SINGULAR(0.000000000001) HCONVERGE(0, ABSOLUTE) LCONVERGE(0, ABSOLUTE) PCONVERGE(0.000001, ABSOLUTE)

/FIXED=age gender education race_d1 race_d2 race_d3 hisp time covid2020 covid2021 covid20_hisp covid21_hisp | SSTYPE(3)

/METHOD=ML

/PRINT=DESCRIPTIVES SOLUTION TESTCOV

/RANDOM=INTERCEPT time | SUBJECT(uasid) COVTYPE(VC).

MIXED C WITH age gender education race_d1 race_d2 race_d3 hisp time covid2020 covid2021 covid20_hisp covid21_hisp

/CRITERIA=DFMETHOD(SATTERTHWAITE) CIN(95) MXITER(100) MXSTEP(10) SCORING(1)

SINGULAR(0.000000000001) HCONVERGE(0, ABSOLUTE) LCONVERGE(0, ABSOLUTE) PCONVERGE(0.000001, ABSOLUTE)

/FIXED=age gender education race_d1 race_d2 race_d3 hisp time covid2020 covid2021 covid20_hisp covid21_hisp | SSTYPE(3)

/METHOD=ML

/PRINT=DESCRIPTIVES SOLUTION TESTCOV

/RANDOM=INTERCEPT time | SUBJECT(uasid) COVTYPE(VC).

**Interactions with education**

MIXED N WITH age gender race_d1 race_d2 race_d3 Hisp educ_cent time covid2020 covid2021 covid20_educ covid21_educ

/CRITERIA=DFMETHOD(SATTERTHWAITE) CIN(95) MXITER(100) MXSTEP(10) SCORING(1)

SINGULAR(0.000000000001) HCONVERGE(0, ABSOLUTE) LCONVERGE(0, ABSOLUTE) PCONVERGE(0.000001, ABSOLUTE)

/FIXED=age gender race_d1 race_d2 race_d3 Hisp educ_cent time covid2020 covid2021 covid20_educ covid21_educ | SSTYPE(3)

/METHOD=ML

/PRINT=DESCRIPTIVES SOLUTION TESTCOV

/RANDOM=INTERCEPT time | SUBJECT(uasid) COVTYPE(VC).

MIXED E WITH age gender race_d1 race_d2 race_d3 Hisp educ_cent time covid2020 covid2021 covid20_educ covid21_educ

/CRITERIA=DFMETHOD(SATTERTHWAITE) CIN(95) MXITER(100) MXSTEP(10) SCORING(1)

SINGULAR(0.000000000001) HCONVERGE(0, ABSOLUTE) LCONVERGE(0, ABSOLUTE) PCONVERGE(0.000001, ABSOLUTE)

/FIXED=age gender race_d1 race_d2 race_d3 Hisp educ_cent time covid2020 covid2021 covid20_educ covid21_educ | SSTYPE(3)

/METHOD=ML

/PRINT=DESCRIPTIVES SOLUTION TESTCOV

/RANDOM=INTERCEPT time | SUBJECT(uasid) COVTYPE(VC).

MIXED O WITH age gender race_d1 race_d2 race_d3 Hisp educ_cent time covid2020 covid2021 covid20_educ covid21_educ

/CRITERIA=DFMETHOD(SATTERTHWAITE) CIN(95) MXITER(100) MXSTEP(10) SCORING(1)

SINGULAR(0.000000000001) HCONVERGE(0, ABSOLUTE) LCONVERGE(0, ABSOLUTE) PCONVERGE(0.000001, ABSOLUTE)

/FIXED=age gender race_d1 race_d2 race_d3 Hisp educ_cent time covid2020 covid2021 covid20_educ covid21_educ | SSTYPE(3)

/METHOD=ML

/PRINT=DESCRIPTIVES SOLUTION TESTCOV

/RANDOM=INTERCEPT time | SUBJECT(uasid) COVTYPE(VC).

MIXED A WITH age gender race_d1 race_d2 race_d3 Hisp educ_cent time covid2020 covid2021 covid20_educ covid21_educ

/CRITERIA=DFMETHOD(SATTERTHWAITE) CIN(95) MXITER(100) MXSTEP(10) SCORING(1)

SINGULAR(0.000000000001) HCONVERGE(0, ABSOLUTE) LCONVERGE(0, ABSOLUTE) PCONVERGE(0.000001, ABSOLUTE)

/FIXED=age gender race_d1 race_d2 race_d3 Hisp educ_cent time covid2020 covid2021 covid20_educ covid21_educ | SSTYPE(3)

/METHOD=ML

/PRINT=DESCRIPTIVES SOLUTION TESTCOV

/RANDOM=INTERCEPT time | SUBJECT(uasid) COVTYPE(VC).

MIXED C WITH age gender race_d1 race_d2 race_d3 Hisp educ_cent time covid2020 covid2021 covid20_educ covid21_educ

/CRITERIA=DFMETHOD(SATTERTHWAITE) CIN(95) MXITER(100) MXSTEP(10) SCORING(1)

SINGULAR(0.000000000001) HCONVERGE(0, ABSOLUTE) LCONVERGE(0, ABSOLUTE) PCONVERGE(0.000001, ABSOLUTE)

/FIXED=age gender race_d1 race_d2 race_d3 Hisp educ_cent time covid2020 covid2021 covid20_educ covid21_educ | SSTYPE(3)

/METHOD=ML

/PRINT=DESCRIPTIVES SOLUTION TESTCOV

/RANDOM=INTERCEPT time | SUBJECT(uasid) COVTYPE(VC).
